# The Role of NLRP3 Inflammasome in the Pathogenesis of Traumatic Brain Injury

**DOI:** 10.3390/ijms21176204

**Published:** 2020-08-27

**Authors:** Natasha Irrera, Massimo Russo, Giovanni Pallio, Alessandra Bitto, Federica Mannino, Letteria Minutoli, Domenica Altavilla, Francesco Squadrito

**Affiliations:** 1 Department of Clinical and Experimental Medicine, University of Messina, c/o AOU Policlinico G. Martino, Via C. Valeria Gazzi, 98,125 Messina, Italy; nirrera@unime.it (N.I.); russom@unime.it (M.R.); gpallio@unime.it (G.P.); abitto@unime.it (A.B.); fmannino@unime.it (F.M.); lminutoli@unime.it (L.M.); 2 Department of Biomedical, Dental, Morphologic and Functional Imaging Sciences, University of Messina, c/o AOU Policlinico G. Martino, Via C. Valeria Gazzi, 98,125 Messina, Italy; daltavilla@unime.it

**Keywords:** traumatic brain injury, NLRP3 inflammasome, neuroinflammation, pyroptosis, SARS-CoV-2

## Abstract

Traumatic brain injury (TBI) represents an important problem of global health. The damage related to TBI is first due to the direct injury and then to a secondary phase in which neuroinflammation plays a key role. NLRP3 inflammasome is a component of the innate immune response and different diseases, such as neurodegenerative diseases, are characterized by NLRP3 activation. This review aims to describe NLRP3 inflammasome and the consequences related to its activation following TBI. NLRP3, caspase-1, IL-1β, and IL-18 are significantly upregulated after TBI, therefore, the use of nonspecific, but mostly specific NLRP3 inhibitors is useful to ameliorate the damage post-TBI characterized by neuroinflammation. Moreover, NLRP3 and the molecules associated with its activation may be considered as biomarkers and predictive factors for other neurodegenerative diseases consequent to TBI. Complications such as continuous stimuli or viral infections, such as the SARS-CoV-2 infection, may worsen the prognosis of TBI, altering the immune response and increasing the neuroinflammatory processes related to NLRP3, whose activation occurs both in TBI and in SARS-CoV-2 infection. This review points out the role of NLRP3 in TBI and highlights the hypothesis that NLRP3 may be considered as a potential therapeutic target for the management of neuroinflammation in TBI.

## 1. Traumatic Brain Injury (TBI)

Traumatic brain injury (TBI) is one of the most common cause of disability and mortality worldwide both in children and adolescents and may have a strong correlation with neurodegenerative diseases such as Parkinson’s and Alzheimer’s disease [1,2]. TBI is an acquired brain injury (ABI), not hereditary neither congenital or induced by a birth trauma which may be recognized as mild, moderate or severe following the application of the Glasgow coma scale (GCS) [3,4]. The parameters evaluated in the GCS are motor performance, verbal responses and eye opening: the obtained total score may indicate a severe (3–8), moderate (9–12) or mild TBI (13–15). Most TBI cases are mild and very often the symptomology represented by headache, dizziness, and confusion is self-limiting and disappear in a few days without sequelae. Repetitive episodes of mild TBI—as happens to professional athletes or military workers—may potentially cause the development of a chronic neuroinflammatory condition that causing brain scars may worsen patient outcomes and result in long-term disability [5,6,7].

TBI affects the population worldwide with an important impact not only on the quality of life of patients, but also on the number of hospitalization and on the annual cost for the healthcare system. In particular, epidemiological data showed that TBI occurs in 69 million individuals each year and South East Asian and Western Pacific area are particularly affected [8]. Of the 1.7 million people suffering TBI in the United States most of them are adolescents (aged 15 to 19 years) and adults (aged 65 years and older) with an incidence of approximately 500 in 100,000 per year. The Center for Disease Control and Prevention (CDC) has estimated that about 1.5 million people survive a TBI, thus causing about 230,000 hospitalization in the US [9]. According to the available data, in 2014 TBI caused 288,000 hospitalizations and 56,800 deaths both in adults and in children with a direct annual cost of $331.1 million [10,11]; in 2015, the number of hospitalizations reached 344,030 [9] and the following year TBI cases were 55.5 million [12]: these numbers demonstrate that TBI represents an important problem of public health that every year has a negative impact on several socioeconomic aspects of our community.

The most common causes of death related to TBI are due to intentional self-harm (32.5%), unintentional falls (28.1%) and motor vehicle crashes (18.7%). In particular, falls and road injuries represent the main cause of non-fatal cases of TBI, reflecting the 2016 data obtained from the Global Burden of Diseases, Injuries, and Risk Factors (GBD) [12]. More than 73% of TBI cases affect male population according to the TBI Model System National Database Statistics from 2017, even if the frequency is equivalent in males and females with an age > 65 [13].

This review will provide a brief overview of the pathophysiology of TBI and in particular aims to describe the role played by a complex of proteins known as the nucleotide-binding oligomerization domain-like receptor pyrin domain-containing-3 (NLRP3) inflammasome, in the neuroinflammatory response in TBI, thus highlighting the hypothesis that NLRP3 may be considered as a potential therapeutic target for the management of neuroinflammation in TBI.

Highlights:

(1) TBI is one of the most common cause of disability and mortality worldwide;
(2) TBI affects the population worldwide with an important impact on i) the quality of life of patients, ii) the number of hospitalizations and iii) the annual cost of the healthcare;
(3) This review will provide a brief overview of the pathophysiology of TBI and aims to describe the role of the NLRP3 inflammasome in TBI.

## 2. Pathophysiology of TBI

Cell membrane disruption due to traumatic brain injury is responsible for the alteration of ions and neurotransmitters equilibrium that modify the membrane potential. The acute phase of TBI, within an hour after injury, is characterized by a significant release of glutamate from presynaptic terminal that disrupts ionic balance on postsynaptic membranes. Glutamate release is responsible for excitotoxicity, a process that contributes to the pathophysiology of TBI, basically characterized by the increase of glutamate and other neurotransmitters that stimulating NMDA (N-methyl-d-aspartate) and AMPA (amino-3-hydroxy-5-methyl-4-isoxazolepropionic acid) receptors [14] causing intracellular accumulation of calcium, overproduction of nitric oxide (NO) with consequent formation of free radicals and oxidative stress that disrupting membranes causes DNA damage and promotes pro-death signals [15]. Among the proapoptotic signals, p53 expression is markedly increased following glutamate receptor stimulus and induces neuronal injury and death through apoptosis and autophagy [16]. Apoptotic neurons show DNA degradation and caspases activation whereas necrotic cells show membrane disruption, cell swelling and cytoplasm vacuolization: excitotoxic cell death may happen through a mix of apoptosis and necrosis [17]. Autophagic cell death represents a survival strategy in response to stress and is involved in the excitotoxic cell death mechanisms through lysosomes formation [18].

Excitotoxicity may also be independent from glutamate and is mainly due to the glutamate-independent opening of NMDA receptors since the unexpected movements of the head activate neuronal membranes mechanoporation, eventually activating ischemic events, intracellular calcium accumulation and cell death mechanisms following TBI [19,20]. As a matter of fact, NMDA receptors respond to mechanical stress following TBI and GluN2B subunit is considered as a mediator of the mechanosensitive responses, thus activating pro-death signals. This mechanism is strictly linked to the presence of glutamate, in fact, the consequent calcium influx is inhibited if glutamate binding sites are occupied by specific blockers [21]. On the contrary, Maneshi et al. showed that mechanical stress may cause NMDA receptor activation in absence of glutamate, thus demonstrating that NMDA receptors may be activated following a mechanical stimulus and independently of glutamate [22]. NMDA receptors may be also stimulated by other ligands, such as aspartate in association with glycine or D-serine, even if the affinity of glutamate is higher than other molecules [23]. This is an important molecular aspect in TBI because aspartate significantly increases following TBI, thus contributing to excitotoxicity activation in an independent fashion from glutamate [24]. TBI causes severe alterations in cell membranes, promoting calcium influx, and calcium may also directly induce excitotoxicity as a consequence of TBI. Moreover, calcium influx may be promoted following ischemic processes related to TBI [19]. In particular, the anaerobic environment after ischemia activates sodium–calcium exchangers (NCX), acid-sensing ion channels (ASIC) and transient receptor potential channels (TRPM), thus increasing intracellular calcium levels and consequently activating excitotoxic pathways and cell death processes independently of glutamate [25,26,27,28]. Calcium is generally transported in the endoplasmic reticulum by a pump called SERCA (sarcoplasmic/endoplasmic reticulum calcium-ATPase); its activity is based on the exchange of two calcium ions for every ATP molecule [29], therefore, SERCA may contribute to excitotoxicity, increasing cytosolic calcium concentration through ryanodine receptors (RyR) and inositol-1,4,5-trisphosphate receptors RyR (IP3R) which are activated following TBI [30,31].

Accumulation of intracellular Ca^2+^ stimulates Ca^2+^ uptake in mitochondria, thus inducing oxidative stress, impairing mitochondrial and even cognitive function [32,33]. Ionic and neurotransmitter alterations after TBI compromise several cellular functions, including glucose metabolism, free radical formation and redox balance [34]. Cerebral glucose metabolism (CMRglc) changes have been observed within eight h following TBI, probably due to an increased request of energy to balance ionic alterations and neuronal membrane potential [35,36]. After the increase of CMRglc, its decrease has been detected both in experimental in vivo models and in humans [37,38]. The reason of the increase/decrease switch is still unknown, but it could be due to (i) the reduction of blood availability, (ii) the presence of glucose transporters defects, (iii) the decrease of metabolic glucose request [34]. Metabolic alterations together with ionic and neurotransmitter imbalance, free radical formation (increased production of O2− and hydroxyl radical (^•^OH)) and oxidative stress activation worsens the prognosis of TBI, thus inducing cell membrane disruption, protein/DNA damage and cognitive impairment.

Highlights:

(1) Excitotoxicity is a process that contributes to the pathophysiology of TBI with an increase of neurotransmitters and glutamate levels;
(2) TBI can induce glutamate-independent excitotoxicity, stimulating the release of calcium;
(3) The increased levels of ions and glutamate cause DNA damage, oxidative stress activation, proapoptotic signals.

## 3. TBI and Neuroinflammation

TBI is primarily the consequence of a direct damage to the brain, but secondary events may occur, such as inflammation, edema, oxidative–nitrosative stress and activation of cell death mechanisms. As afore mentioned, all these secondary processes influence patient outcome and recovery [39,40]. Therefore, TBI is characterized by two phases: a primary injury characterized by a rapid damage, often mechanical and a secondary and delayed injury which occurs in minutes, hours, months up to years [41]. The first phase is characterized by Blood Brain Barrier (BBB) alteration and disruption, blood flow reduction and a direct damage to neuronal and glial cells. Neuroinflammation plays a central role in the secondary phase and causes cell degeneration and alteration of neural/synaptic transmission and plasticity [42,43,44,45,46]. Neuroinflammation is a very complex event and is first of all related to the passage of peripheral immune mediators through the BBB followed by microglial and peripheral neutrophil activation, lymphocytes and monocyte-derived macrophages infiltration, proinflammatory and anti-inflammatory cytokines release and immune cells recruitment [47,48]. Microglial activation induces the production of proinflammatory cytokines such as Tumor Necrosis Factor alpha (TNF-α), interleukin (IL)-1β and IL-6 [49,50]; if on one hand activated microglia and neuroinflammation may have a neuroprotective role, on the other hand an exaggerated activation with a consequent cytokine storm production may contribute to neurological symptoms and neurodegeneration [51].

In the acute phase of TBI there is not only a production and release of proinflammatory cytokines, but also of anti-inflammatory mediators, such as IL-4 and IL-10 [52,53]. This was observed also in clinical trials where IL-6, IL-1β and IL-8 increased 48–72 h after TBI whereas high levels of the anti-inflammatory IL-10 were observed up to 5 days later, in patients with severe TBI [54,55]. Many efforts have been made to find a possible therapeutic approach by targeting secondary injury processes, including calcium channel blockers, corticosteroids, excitatory amino acid inhibitors, NMDA receptor antagonist, free radical scavengers, magnesium sulfate and growth factors [56]. In particular, several compounds showed interesting anti-inflammatory effects, reducing (i) proinflammatory cytokines, (ii) microglial activation and (iii) some transcription factors and receptors involved in neuroinflammation, such as Nuclear factor kappa B (NF-κB) and Toll-like receptor-4 (TLR4) [57] (Table 1). Many of these strategies provided positive effects in reducing neuroinflammation in experimental models of TBI, but failed when tested in clinical trials, therefore, no effective therapy currently exists for the treatment of neuroinflammation. However, a Phase II randomized control trial showed that the use of recombinant human IL-1 receptor antagonists was safe in the human severe TBI population and has been already used in the clinical practice [58,59].

Membrane degeneration and the exaggerate production of Reactive Oxygen Species (ROS) by glial cells provokes lipid peroxidation, ionic imbalance and consequent ATP discharge, thus promoting cell necrosis and accelerating neurodegenerative processes [60]. ATP released by altered membranes or necrotic cells may, in turn, bind the purine P2X7 receptors thus contributing to the inflammatory process during TBI: in fact, their activation is considered as an upstream signal of the NOD-, LRR- and pyrin domain-containing 3 (NLRP3) inflammasome [61,62]. The NLRP3 inflammasome which further triggers neuroinflammatory processes is not only activated by ATP, but also by the Damage-associated molecular patterns (DAMPs) which are released following cell disruption resulting from the mechanical and/or the secondary injury in TBI [47,63]. The proinflammatory cytokines TNF-α, IL-1β and IL-6 are further upregulated by microglial activation in response to DAMPs, thus worsening the inflammatory reaction and stimulating cell death mechanisms related to TBI. In particular, TNF-α acts as one of the main actors in promoting cell necrosis and in turn, again, membranes are broken up and DAMPs are released, thus amplifying neuroinflammation and cell death mechanisms [43].

Highlights:

(1) TBI is primarily the consequence of a direct damage to the brain;
(2) Neuroinflammation plays a central role in the secondary phase of TBI;
(3) Microglial activation induces the release of proinflammatory cytokines worsening neuroinflammation;
(4) Glial cells stimulate ROS release which causes lipid peroxidation, ionic imbalance and ATP discharge, promoting cell necrosis and accelerating neurodegenerative processes;
(5) ATP binds purine P2X7 receptors which represent an upstream signal of the NLRP3 inflammasome.

## 4. NLRP3 Inflammasome

DAMPs may mediate cytokines production through different mechanisms and the activation of the inflammasome complex is one of those. Inflammasomes are differently distributed in the brain: NLRP3 is mainly located in microglia, but it was also found in oligodendrocytes following dexamethasone stimulation and in astrocytes [64,65,66], whereas NLRP1 and AIM2 are expressed in neurons [67].

NLRP1 inflammasome represents one of the main components involved in the primary inflammatory response to TBI. In fact, high levels of potassium (K+) ions released following TBI stimulate pannexin-1 channels which, in turn, activate NLRP1 inflammasome. NLRP1 activation mediates X-linked inhibitor of apoptosis protein (XIAP) cleavage, thus inducing caspase-1 and caspase-3 cleavage. The proteolytic activation of caspase-1 causes IL-1β and IL-18 release whereas caspase-3 activation determines DNA fragmentation and apoptosis induction [68,69]. An experimental animal model that exploited NLRP1 knockout mice showed that the lack of NLRP1 did not improve the damages caused by a controlled cortical impact (CCI) injury, thus demonstrating its nonessential role, at least in this model of TBI [70]. However, the role of the NLRP1 inflammasome needs to be better investigated in order to design an effective therapeutic approach.

**Figure 1:**
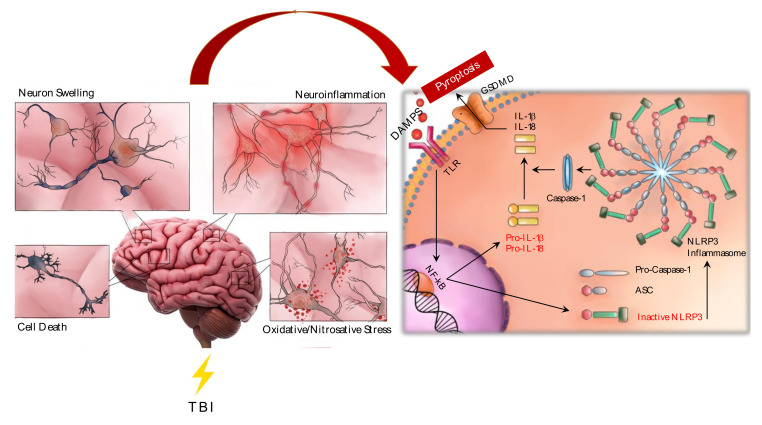
TBI (Traumatic brain injury) activates different processes such as edema, oxidative stress, cell death mechanisms and inflammation. In the secondary phase, neuroinflammation plays a key role and different proinflammatory cytokines are released such as TNF-α (Tumor Necrosis Factor alpha), (interleukin) IL-6, IL-1β and last, but not least (NOD-, LRR- and pyrin domain-containing 3) NLRP3 inflammasome is activated. NLRP3 inflammasome stimulation mediates the release of caspase-1, IL-1β and IL-18. Moreover, NLRP3 activates the pyroptosis as a mechanism of cell death.

In the last years, the attention of the researchers is focused on the role of the NLRP3 inflammasome in several brain diseases, including Alzheimer disease (AD), Parkinson’s disease (PD), amyotrophic lateral sclerosis (ALS), TBI and central nervous system (CNS) infection. The activation of the NLRP3 inflammasome may also represent the priming step caused by innate immune sensors, the NOD-like receptors (NLRs) [71,72]. NLRs are cytosolic pattern recognition receptors composed by a sensor molecule, an adaptor protein and an effector component, that once activated, form the inflammasome complex [73]. The sensor molecule of the NLRP3 inflammasome is NLRP3, the adaptor protein is ASC, also known as PYCARD, whereas caspase-1 represents the effector protein [74]. NLRP3 consists of an amino-terminal pyrin domain (PYD), a central NACHT domain and a carboxy-terminal leucine-rich repeat domain (LRR domain). When NLRP3 is activated following a specific stimulus, the inflammasome oligomerizes and recruits ASC protein. The ASC domain mediates the activation of the inactive procaspase 1 into its active form, caspase-1, thus forming the complete NLRP3 inflammasome. The active caspase-1, in turn, facilitates the conversion of the pro-IL-1β and pro-IL-18 into their active form, IL-1β and IL-18 [75,76] (Figure 1). The production of the proinflammatory cytokines through NLRP3 activation during TBI creates an inflammatory environment that worsens the damage associated with TBI, thus worsening the prognosis of the disease. High levels of ASC, caspase-1 and IL-18 were detected in the serum of patients with a diagnosis of severe TBI, therefore, it has been speculated a possible use as biomarkers [77,78]. Both IL-1β and IL-18 promote the accumulation of ROS which, in turn acting as DAMPs, may stimulate both caspase-1 and NLRP3 inflammasome activation, with a further production of IL-18 and apoptosis activation, thus causing a feedback loop mechanism between oxidative stress and NLRP3 inflammasome activation [79,80]. The exaggerated and continuous NLRP3 inflammasome stimulation may lead to cell death through the activation of the pyroptosis mechanism [81]. However, surprising unpublished data from our lab demonstrated that NLRP3 overexpressing mice did not display exaggerated signs of neuroinflammation nor increased mortality rate following moderate TBI obtained through a CCI procedure, suggesting that despite the marked increase of DAMPs in this setting an overexpression of NLRP3 is not detrimental by itself.

Highlights:

(1) DAMPS mediate inflammasome activation;
(2) NLRP1 inflammasome stimulation induces caspase-1/caspase-3 cleavage and consequently IL-1β/IL-18 release and apoptosis activation;
(3) NLRP3 inflammasome activates caspase-1 which, in turn, promotes IL-1β/IL-18 release;
(4) Both IL-1β and IL-18 promote ROS accumulation;
(5) ROS may stimulate both caspase-1 and NLRP3 inflammasome, with a further production of IL-18 and apoptosis activation;
(6) NLRP3 inflammasome activation induces pyroptosis.

## 5. NLRP3 Inflammasome Regulation

The exact mechanism of NLRP3 activation is still unclear, but different regulatory mechanisms and molecules may positively or negatively modulate NLRP3 inflammasome. A key factor in its regulation seems to be played by NIMA-related kinase 7 (NEK7), a Ser/Thr mitotic kinase that is recruited for the formation of the NLRP3 inflammasome complex. In an experimental setting NEK7 downregulation ameliorated neurological deficits, reduced NLRP3 inflammasome activation and cell death mechanism, including pyroptosis [82]. NEK7 has been found in NLRP3/ASC complexes: ASC complexes require NLRP3 complex formation and the interaction between NEK7 and NLRP3 is essential [83]. A recent study demonstrated the important role of NEK7 in regulating NLRP3 inflammasome, in fact, NEK7 knockdown significantly decreased caspase-1 activation, thus inhibiting pyroptosis and downstream inflammation following TBI [84]. However, the whole mechanism that explains how NEK7 modulates NLPRP3 inflammasome in neurons is still unknown. The NLRP3/ASC complex formation is not only dependent on NEK7 but may be also promoted by β-catenin, which is often activated by Wnt proteins, suggesting that Wnt/β-catenin signal may also be involved in the NLRP3 inflammasome activation [85]. Canonical Wnt/β-catenin pathway is widely involved in cell and tissue development and regeneration [86]; when Wnt proteins bind with their specific Frizzled receptors, β-catenin migrates into the nucleus and promotes the transcription of its target genes. However, β-catenin may be responsible for NLRP3 inflammasome activation apart from its activity at transcriptional level, in fact, Huang L. et al. demonstrated that β-catenin did not stimulate neither NLRP3 protein nor mRNA expression but promoted NLRP3 inflammasome assembly with ASC to obtain the active NLRP3 complex [85]. In contrast, a previous study showed that β-catenin activation may increase NLRP3 expression [87], even if transcriptional factors may have effects independent of transcriptional activity [88]; however, these findings point out a role for this pathway in the activation of NLRP3 cascade.

Different regulators may positively modulate NLRP3 inflammasome such as DDX3X, GBP5 and cathepsin, thus inducing NLRP3 oligomerization and promoting ASC assembly [89,90,91]. On the other hand, HSP70, PRDX1, NLRC3, SHP and POPs may negatively modulate NLRP3 assembly, acting on NLRP3 itself or inhibiting the formation of NLRP3/ASC complex or the interaction between ASC and procaspase-1 [92,93,94,95,96]. Post-translational modification may also promote NLRP3 assembly and interaction, in fact, the dephosphorylation of pyrin domain mediated by PP2A significantly inhibited NLRP3 inflammasome activation thus blocking ASC assembly that requires pyrin domain (PYD)–PYD interactions [97].

Highlights:

(1) NEK7 is recruited for the formation of the NLRP3 inflammasome complex;
(2) The NLRP3/ASC complex formation may be induced by β-catenin;
(3) DDX3X, GBP5 and cathepsin positively modulate NLRP3 inflammasome;
(4) HSP70, PRDX1, NLRC3, SHP and POPs negatively modulate NLRP3 assembly.

## 6. NLRP3 and Oxidative Stress

Oxidative stress is one of the mechanisms involved in the secondary injury following TBI, with the accumulation of nitrogen and reactive oxygen species [98].

NADPH oxidases (NOX) are a group of transmembrane enzymes that transporting an electron from cytosolic NADPH to reduce oxygen play the main role of producing ROS [99]. Although NOX are involved in different pathways and in the immune response, the continuous activation of NOX significantly contributes to the damage in neurodegenerative conditions, including TBI [100,101]. In particular, NOX2 expression rapidly increases one hour following TBI, while other isoforms peaks at 24–96 h [102,103]. What is particularly relevant is that neurons are the cells primarily involved in the first peak, whereas, at a later stage, microglial cells are involved in NOX2 increase, also 7 and 28 days, and even one year after TBI [104,105]. The repeated and chronic microglial activation exacerbates the primary damage of the injury, worsens the outcomes and delays the recovery.

NOX3 and NOX4 have also been reported to be augmented following TBI in experimental animal models and a correlation to the increased expression of NOX2 and NOX4 with TBI severity was observed [106]. It is clear that NOX isoforms represent an important source of intracellular and extracellular ROS for NLRP3 inflammasome activation. In particular, NOX2-derived oxidative stress contributes to NLRP3 inflammasome stimulation through TXNIP interaction with NLRP3 following TBI, thus increasing caspase-1 and IL-1β [107]. Therefore, targeting NOX2 may be useful for the management of TBI in order to reduce oxidative stress and to avoid NLRP3 activation.

Highlights:

(1) Oxidative stress processes are activated in the secondary phase of TBI;
(2) NADPH oxidases (NOX) are a group of enzymes that contributes to ROS release;
(3) NOX isoforms represent an important source of ROS for NLRP3 inflammasome activation.

## 7. NLRP3 and Pyroptosis

NLRP3 inflammasome activation stimulates a mechanism of cell death called pyroptosis which is rapid and activated in response to inflammatory stimuli. Pyroptosis is different from the other programmed cell death and is basically characterized by cell swelling, pores formation on plasma membranes and release of the proinflammatory cytokines IL-1β and IL-18 [108,109]. The activation of the “canonical” pyroptosis requires caspase-1, resulting from NLRP3 inflammasome activation, which cleaves the carboxy-terminal domain of gasdermin D (GSDMD), the key mediator of pyroptosis [110,111]. The amino-terminal domain of GSDMD mediates the cell death mechanism by forming a pore in the plasma membrane and, in addition, activates the NLRP3 inflammasome [112,113]. Pyroptosis activation through GSDMD causes cell death, but also stimulates the release of IL-1β and IL-18, thus increasing the downstream interleukins already augmented following NLRP3 stimulation [114]. The noncanonical pyroptosis, which involves caspase-4/5/11 may be activated following TBI, however, the canonical pyroptosis seems to be the main pathway in CNS injuries and in TBI, following NLRP3 recruitment [115]. Further studies are needed to elucidate pyroptosis and its related consequences not only to understand the pathophysiology of TBI, but also to develop new therapeutic strategies.

Highlights:

(1) NLRP3 inflammasome activation stimulates pyroptosis;
(2) The activation of the “canonical” pyroptosis recruits caspase-1 resulting from NLRP3 activation;
(3) caspase-1 cleaves gasdermin D (GSDMD) which represents the key mediator of pyroptosis;
(4) Pyroptosis promotes the release of IL-1β and IL-18, further increasing proinflammatory cytokines levels;
(5) The canonical pyroptosis is activated in TBI following NLRP3 recruitment.

## 8. NLRP3 Activation in Experimental Models of TBI and in Patients

The role of the NLRP3 inflammasome in post-traumatic neuroinflammation was demonstrated in experimental in vivo models of TBI and also in humans affected by a moderate/severe TBI. Liu et al. demonstrated that NLRP3 inflammasome, ASC and caspase-1 mRNA expression was increased six h following a fluid percussion injury and NLRP3 and caspase-1 protein levels were still increased in brains 24 h after injury [64]. NLRP3, caspase-1 and IL-1β expression was also significantly increased in rats 12 h and 24 h after a blast-induced traumatic brain injury and in mice subjected to an experimental model of cold brain injury [116,117].

Chen Y et al. showed that NLRP3 mRNA expression started to become high within the first six h post-TBI and reached the peak at 24 hours. Following this peak, NLRP3 expression declined to reach another peak three days post-TBI; at day seven, NLRP3 was reduced, but its expression was always higher than in controls [118]. In the same study, the authors demonstrated that NLRP3, IL-1β, IL-18 and caspase-1 levels were significantly increased in human brain specimens collected from patients with severe TBI, thus confirming that the traumatic injury activates NLRP3 inflammasome with the consequent increase of the proinflammatory cytokine production in human brains.

Interestingly, NLRP3 increased levels were also observed in the cerebrospinal fluid (CSF) of children with severe TBI. In particular, an association between an age ≤ four years and NLRP3 high levels was found. This data is interesting because pediatric patients usually have better outcomes than adults even if children with an age ≤ four years have a worse outcome [119]. In fact, neuroinflammation was strongly observed in young children, thus worsening their outcomes [120,121]. Previous studies have demonstrated an increased expression of IL-1β in the CSF as a consequence of NLRP3 activation both in adults and in children following TBI [122,123,124]. Fascinatingly, NLRP3 increase changes in relation to time: NLRP3 levels were high 24 h post-TBI, lower after 48 h and increased three or four days after injury [119]. The data described so far (Table 2) indicated that NLRP3 activation is first of all the consequence of the primary injury and plays a key role in TBI, thus influencing the prognosis of patients.

Highlights:

(1) NLRP3 inflammasome, caspase-1 and IL-1 β expression was observed in animal models of TBI at different time points.
(2) Increased levels of NLRP3, IL-1β, IL-18 and caspase-1 were also observed in patients following TBI.

## 9. NLRP3 as a Biomarker for TBI Progression in CTE and Other Neurodegenerative Diseases

In the last few years, the role of neuroinflammation in the pathogenesis of neurodegenerative diseases is resulted to be crucial. The neuroinflammatory process caused by TBI, even after mild TBI, may be considered as a risk factor for other neurodegenerative diseases such as ALS, AD and PD.

In fact, it has been demonstrated that neuroinflammation established after repeated TBIs plays a key role in the pathogenesis of chronic traumatic encephalopathy (CTE), a progressive neurodegenerative disease recurring in sport players exposed to repeated concussions [125].

An extensive search for relevant biomarkers as well as for reliable diagnostic and prognostic biomarkers in the preclinical stage of AD, ALS and PD has been carried out to help physicians to identify patients at high risk of developing TBI complication, but the results obtained are still unsatisfactory. Prognostic biomarkers may reduce phenotypic heterogeneity and improve statistical power for a fixed sample size. Moreover, pharmacodynamic biomarkers may help to demonstrate the presence of the intended biologic effect [126,127]. The data discussed so far suggest that NLRP3 inflammasome and the molecules released following its activation may be considered as potential biomarkers to improve the early detection of TBI complications as well as the preclinical stage of different neurodegenerative diseases.

Highlights:

(1) Neuroinflammation caused by TBI may be considered as a risk factor for neurodegenerative diseases, such as ALS, AD, PD and CTE;
(2) NLRP3 inflammasome and the molecules released following its activation may be considered as potential biomarkers of neurodegenerative diseases.

## 10. NLRP3 Inflammasome and Therapeutic Approaches

The discovery of the important role played by NLRP3 inflammasome in TBI has led to the hypothesis that NLRP3 may be considered as an important target to manage neuroinflammation and to improve TBI recovery. In fact, knockout mice for NLRP3 showed a significant reduction of neuroinflammatory processes and a significant improvement of the impaired functional outcomes [128,129]. Moreover, different new therapeutic approaches have been found that directly or indirectly target NLRP3 inflammasome: natural compounds such as mangiferin, omega-3 fatty acids and apocynin; nonspecific NLRP3 inhibitors such as ASC antibodies, the NF-κB inhibitor, BAY 11–7082; specific NLRP3 inhibitors as MCC950 and JC-124; other drugs such as propofol and telmisartan [67,107,116,129,130,131,132,133,134,135,136] (Table 3).

Both the non-selective and selective NLRP3 inhibitors significantly reduced NLRP3 expression and its downstream molecules, thus showing neuroprotective effects and demonstrating the relevance of targeting NLRP3 in TBI. The direct NLRP3 inhibition by using small molecules represents a specific/cost-effective approach and is less invasive than others aiming at reducing or arresting cytokines release, such as IL-1β [75]. CRID3/CP-456773, known as MCC950, is one of the most effective and specific NLRP3 inhibitor: it inhibits both the canonical and noncanonical NLRP3 inflammasome activation, without affecting other inflammasomes such as NLRP1 or AIM2 [130,137]. However, MCC950 seemed to be hepatotoxic so that a clinical trial was interrupted [138]. Other molecules recognized as specific NLRP3 inhibitors that do not affect the other inflammasomes are C172, tranilast and oridonin, but none of these have been tested for the management of traumatic brain injury [139,140,141].

Highlights:

(1) NLRP3 inflammasome may be considered as a therapeutic target;
(2) Different therapeutic approaches may directly or indirectly target NLRP3 inflammasome;
(3) Both the non-selective and selective NLRP3 inhibitors may reduce NLRP3 expression and its downstream molecules, thus showing neuroprotective effects and demonstrating the relevance of targeting NLRP3 in TBI.

## 11. NLRP3 Inflammasome, SARS-CoV-2 and Possible Consequences in TBI

Complications such as continuous stimuli or infections, such as viral infections, may worsen the prognosis of TBI, altering the immune response and increasing the related neuroinflammatory processes. Viral infections are often related to inflammation and virus proteins, as viroporins, may play a pivotal role in promoting viral infection [142]. Previous studies showed that viroporins promote NLRP3 inflammasome activation [142,143], in fact NLRP3 activation was observed following influenza A virus and SARS-CoV infections [144]. In the past few months and until now, a novel coronavirus, the SARS-CoV-2, is affecting the population worldwide, thus causing a high number of deaths so that the World Health Organization (WHO) has declared coronavirus disease 2019 (COVID-19) as global pandemic. SARS-CoV-2 is an enveloped and positive-strand RNA virus that mainly affects the respiratory tract and is responsible for severe acute respiratory failures that may worsen and evolve in adult respiratory distress syndrome (ARDS), organ failure and death [145,146,147]. SARS-CoV-2 binds the angiotensin-converting enzyme 2 (ACE2) receptor which is expressed in different organs and also in brain [148,149]. One of the main features of COVID-19 is the production of a cytokine storm that worsens the prognosis and the recovery of patients. IL-1β and IL-18 were found in the plasma of patients affected by COVID-19 and it is also probably related to NLRP3 inflammasome activation through virus proteins such as protein E and 3a [150,151]. Both NLRP3-dependent and independent cytokine storm causes severe systemic inflammation that may be often fatal. SARS-CoV-2 may invade the central nervous system as demonstrated by autopsies [152], but no scientific evidences exist so far about the possible persistence of SARS-CoV2 in the brain. However, previous data about others coronaviruses, such as the HCoVOC43RNA, demonstrated that it could be found in the CNS after one year [153]. The reason the virus may persist in the central nervous system is still unknown, but some viruses may remain inactive in brain and may be reactivated under certain conditions [154]. This supposed persistence may have negative long-term consequences also in patients affected by TBI; these patients, if previously infected by SARS-CoV-2, could be more susceptible to neurological manifestations related to the reactivation of the virus that may exacerbate neuroinflammatory processes related to NLRP3 activation.

Highlights:

(1) Viral infections may worsen the prognosis of TBI;
(2) Virus proteins, such as viroporins, may promote NLRP3 inflammasome activation;
(3) IL-1β and IL-18 were found in the plasma of patients affected by COVID-19, probably as a consequence of NLRP3 inflammasome activation;
(4) NLRP3-dependent and–independent cytokines storm causes severe systemic inflammation that may be fatal;
(5) SARS-CoV-2 may invade the central nervous system;
(6) Patients affected by TBI could be more susceptible to neurological manifestations related to SARS-CoV-2 that may stimulate neuroinflammatory processes related to NLRP3 activation.

## 12. Conclusions

TBI represents an important problem of public health, however, an effective therapy has not been found neither to manage the direct damage related to the injury nor to improve the clinical outcomes. Indeed, the management of TBI is not easy to implement in the clinical setting and, as a consequence, it represents a burden for the health care systems in terms of economic cost and future disability. The pathogenesis of TBI is still poorly understood and several molecular pathways are subjected to intense investigation with the aim to clarify the mechanism(s) underlying this devastating clinical condition. Neuroinflammation plays a central role in the second wave of damage that occurs after the initial injury and an intense scientific effort has been spent in the last decades to identify the molecular pathways and the intracellular signal cascade involved in this maladaptive response. Several intracellular events have been explored as potential targets to design innovative strategies and create rationale medicines for treating TBI patients, but the results have been often disappointing and have generated the conclusion that the modulation of neuroinflammation is still far to be accomplished. However, in the last few years the research attention has been drawn to new inflammatory platforms, collectively known as inflammasomes, that have renewed the hope of a possible pharmacological modulation of the exaggerated inflammatory reaction that occurs following traumatic brain injury. The preclinical and clinical evidences so far accumulated are encouraging and suggest that NLRP3 inflammasome may represent an important therapeutic target to manage neuroinflammation and to improve patient outcomes following TBI. Moreover, NLRP3 and the related molecules released following its activation may be useful as potential biomarkers of neuroinflammatory conditions and as predictive factor for other neurodegenerative diseases. Several studies will be needed to further investigate NLRP3 role and to find new strategies to antagonize NLRP3 inflammasome.

## Figures and Tables

**Table 1:** Drugs with anti-inflammatory effects in traumatic brain injury (TBI).

Drugs	Effects on TBI-Related Neuroinflammation (⇓ = Reduction)
Carprofen	⇓ Microglia ⇓ IL-1β ⇓ IL6
Celecoxib	⇓ IL-1β
Indomethacin	
Dexamethasone	⇓ Microglia
Flavopiridol	
Pioglitazone	
Rosiglitazone	
Roscovitine	
Etanercept	⇓ TNF-α
Etazolate	⇓ IL-1β ⇓ Microglia
Erythropoietin	⇓ NF-κB, ⇓ IL-1β ⇓ TNF-α
	⇓ Microglia
Lipoxin A4	⇓ IL-1β, ⇓ IL-6, ⇓ TNFα,
	⇓ Microglia
Minocycline	⇓ Il-1β ⇓ Microglia
N-acetylcysteine	⇓ NF-κB, ⇓ IL-1β⇓ IL-6, ⇓ TNF-α
Progesterone	⇓ IL-6, ⇓ NF-κB
Simvastatin	⇓ TLR4, ⇓ NF-κB⇓ IL-1β, ⇓ TNFα ⇓ IL-6

**Table 2:** Preclinical and clinical evidences related to NLRP3 inflammasome activation in TBI.

References	Preclinical Evidences ( 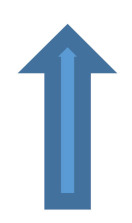 = Increase)	Clinical Evidences ( 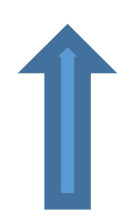 = Increase)
Liu et al. 2013		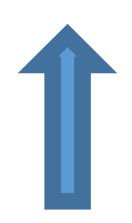 caspase-1
Ma et al. 2016	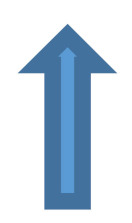 NLRP3, caspase-1 and IL-1β	
Wei et al. 2016	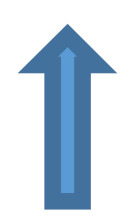 NLRP3, caspase-1 and IL-1β	
Chen et al. 2019		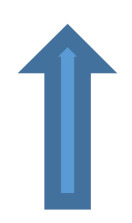 NLRP3, IL-1β 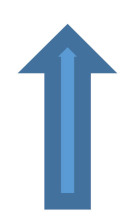 IL-18, caspase-1
Chiaretti et al. 2005		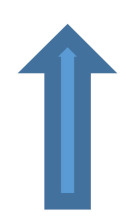 IL-1β, IL-6

**Table 3:** NLRP3 inhibitors in TBI.

Natural Compounds	Nonspecific NLRP3 Inhibitors	Specific NLRP3 Inhibitors	Other Drugs
Mangiferin	ASC antibodies	MCC950	Propofol
Omega-3 fatty acids	NF-κB inhibitor (BAY 11–7082)	JC-124	Telmisartan
Apocynin			

## Abbreviations

DAMPS	Damage-associated molecular patterns
TLR	Toll-like receptor
GSDMD	Gasdermin D
NF- κB	Nuclear factor kappa B
ASC	Apoptosis-associated speck-like protein containing a caspase recruitment domain
